# Is the Metallic Color of Stainless Steel Crown Satisfying for Cooperative Children and their Parents? a Preliminary Study

**DOI:** 10.30476/DENTJODS.2021.91616.1593

**Published:** 2022-12

**Authors:** Fatemeh Moslemi, Amir Mohamad Yasaie, Raziyeh Shojaiepour

**Affiliations:** 1 Dept. of Pediatric Dentistry, School of Dentistry, Kerman University of Medical Sciences, Kerman, Iran; 2 Dept. of Periodontic Dentistry, School of Dentistry, Kerman University of Medical Sciences, Kerman, Iran; 3 Dept. of Pediatric Dentistry, Oral and Dental Diseases Research Center, School of Dentistry, Kerman University of Medical Sciences, Kerman, Iran

**Keywords:** Composite Resins, Color, Patient Satisfaction, Crowns

## Abstract

**Statement of the Problem:**

Currently, the demand for tooth-colored restorations in children and young adults is increasing. Stainless steel crown (SSC) is the most common restoration for decayed primary molars. Given the dark metallic color of SSC, the esthetic appearance of this restoration is poor and subsequently their acceptance is still a matter of debate.

**Purpose:**

This study was conducted to evaluate the effect of restoration’s color on children’s daily living conditions and compare the clinical and radiographic success rates of composite resins with SSC in primary molars.

**Materials and Method:**

This clinical trial study was performed on 70 primary molars in 44 healthy 4- to 7-year-old children. The children were randomly divided into two groups restored with SSC and restored with composite resin. Two researcher-made questionnaires were used to assess the children’s satisfaction concerning the appearance and color of restoration. The data were analyzed with SPSS 20 using chi-squared, Fisher’s, and Mann-Whitney U tests. The significance level was set at p< 0.05.

**Results:**

Children’s satisfaction with restoration color in the treatment session was 75% in the SSC group and 85% in the composite resin group. However, the difference was not statistically significant (p= 0.246). After one year of follow-up, the satisfaction rate decreased to 69% in the SSC group and increased to 90.6% in the composite resin group, with a significant difference (p< 0.001). Moreover, the frequency of clinical success was 95% in the SSC group and 96.7% in the composite resin group, with no statistically significant difference (p= 0.749). The frequency of radiographic success was 87.5% in the SSC group and 100% in the composite resin group; this difference was not significant (p= 0.061).

**Conclusion:**

The results verified that restoration color was not important for cooperative children in the treatment session. However, after one year, children who received composite resin restorations were pointedly more satisfied than those who were treated with SSC restorations.

## Introduction

Aligned and white teeth with a proper contour are essential components of facial beauty and esthetics. Having these criteria, human face would be more attractive which consequently affects individual’s self-confidence and social relationships [ 1 ]. Children are aware of their appearance and beauty of their teeth. According to a deep-rooted view, perception and attention to beauty are completed at eight years of age [ 2 ]. However, new studies in the child psychology field challenge this point of view, and allege that by increasing social media activity, children at the age of 3–5 would be aware of their self-image [ 2 ].

 In dental practice, the acceptance and satisfaction with the color of restoration in children can be influenced by their gender and race [ 3 ] as well as parent’s level of education [ 4 ]. All the time more, parents are looking for esthetic restorations for treatment of their children’s teeth [ 5 ]. The opinions of parents and dentists regarding the ideal treatment can be dissimilar; however, understanding these differences would lead to better communication and appropriate treatment, particularly in children with sensitive parents [ 6 ]. The psychological benefits of improving oral esthetic are a priority over other dental treatments in many individuals [ 7 ] since it has been demonstrated that dental esthetic problems in childhood and adulthood would have a pronounced effect on an individual’s psychological development [ 8 ]. Dental practitioners should provide sufficient information for their patients regarding each possible treatment option and incorporate their desires and needs into their treatment plan since these treatments and their complications would influence their patients' live [ 9 ]. 

The composite resins are esthetic restorations for the crown of primary molars that reduce the need for significant tooth preparation and their retention increase due to their micromechanical bond to the tooth [ 10 ]. However, some of their disadvantages are their restorative failure due to secondary caries and their high technical sensitivity. Therefore, in non-cooperative children or in cases that moisture control is critical, the efficiency of composite resin restoration is potentially low [ 11 ]. 

Despite improvements in the physicochemical properties of dental composite resins, there are significant concerns for their intrinsic toxicity [ 12 ]. Some components of restorative composite resins are released in the oral environment initially during the polymerization reaction and later due to the degradation of the material. In vitro and in vivo studies have clearly shown that these components of restorative composite resins are toxic [ 12 ]. 

Parents and children prefer esthetic restorations over stainless steel crown (SSC) [ 3 ]. SSCs do not satisfy parents concerned about esthetics [ 13 ]. Some parents are even repulsed by the metallic appearance of SSCs [ 2 ]. The age of esthetic perception in children has decreased and SSC restorations cannot provide desired beauty; moreover, limited research have been performed to study the importance of esthetic factors related to the oral cavity in children. Therefore, this study aimed to preliminary study the cooperative children and their parents’ views regarding the color in two groups of composite resin and SSC restoration over one year. In addition, considering the improvements in the properties of composite resin restorative materials and the temporary nature of primary teeth, the study intended to investigate and compare their clinical and radiographic success rate over one-year of follow-up.

## Materials and Method

The present randomized clinical trial study was conducted in 2020 (#IRCT20200831048565N1) with the ethics code (IR.KMU.REC.1398.618( in the Pediatric Department of the School of Dentistry, Kerman University of Medical Sciences. First, the objectives of the study were explained to the children’s parents; they participated in the study after signing informed consent forms.

The inclusion criteria consisted of patients with 4 to 7 years of age, Frankl's behavior rating score of 3 or 4, no history of dental treatment, having at least one vital primary tooth with a carious lesion involving two or more tooth surfaces (one-half to two-thirds of the crown structure remaining(, requiring pulp treatment. The exclusion criteria were patients with systemic conditions, a history of mental disorders, Frankl 1 or 2 levels of cooperation, and non-vital or unrestorable primary molars.

Seventy primary molars with equal extent of caries in several surfaces were restored in 44 children through the following steps. In the first visit, fluoride therapy was performed to get participants be familiar with the dental environment and estimate their cooperation level. The interval between their visits was a maximum of one week. In the next visit, the children were divided into composite resin and SSC groups for restoration based on odd and even numbers. The children’s primary molars were isolated with a rubber dam, followed by pulp therapy (pulpotomy or pulpectomy as needed) by a postgraduate student in pedodontics. 

In the session of restoration, a questionnaire was proposed to assess the parents and children’s satisfaction with the appearance and color of the restoration. Then, the effect of restoration’s color on the child’s daily living conditions at 3-, 6- and 12-month of follow-up was assessed, and the parents’ satisfaction with the appearance of the restored tooth was evaluated through another questionnaire in the 12-month follow-ups.

These two researcher-made questionnaires were based on a previous study [ 14 ]. For evaluation of the questionnaire’s validity, 10 dental specialists reviewed the contents of the questions, which consequently one question was removed, and five questions were reviewed and revised. The validity of the two questionnaires was obtained based on the CVI coefficient at the desired level (0.9). For reliability, 20 children and their parents completed the questionnaire twice with an interval of three weeks (ICC = 0.91).

 The responses were scored as (1) for satisfaction with the color of the restoration or a positive effect on living conditions 🙂, (-1) for dissatisfaction with the color of the restoration or negative impact on living conditions 🙁, and (0) when the color of the teeth was not important for either the child or the parents 😐.

Finally, the clinical and radiographic success rates of the 12-month follow-ups were evaluated.

### Restorative steps in composite resin and SSC groups SSC

After checking the occlusion, the adjacent teeth were separated with a wooden wedge (Mina, Iran). The proximal surfaces were prepared to free the contact with adjacent teeth with a sterile needle bur (Tizkavan, Tehran, Iran) in a high-speed handpiece (NSK, Japan) under water spray. Then, the cusps and occlusal surface were prepared using a sterile bur (Tizkavan, Tehran, Iran) in a high-speed under water spray. A 1-mm space was created with the opposite tooth. The smallest crown size (3M, USA) that completely covered the prepared tooth was selected, contoured, and crimped if necessary and cemented with glass-ionomer (GC-Fuji, Japan).

### Composite resin restoration

After checking the occlusion, complete removal of caries and unsupported enamel was carried out using a sterile fissure bur (Tizkavan, Tehran, Iran) in a high-speed handpiece (NSK, Japan) under water spray. Then a layer of light-cured glass ionomer cement (GC-Fuji, Japan) with a thickness of 1 mm was placed on the reinforced zinc oxide eugenol base (Kemdent, UK) to prevent the eugenol from interfering with the polymerization reaction of the resin and then light-cured with a calibrated light-curing device(Woodpecker, LED-D, China) for 40 seconds. For the second primary molar, a 3M orthodontic band was adapted, and a wedge (Mina, Iran) was placed to secure the band to the tooth. For the first primary molars, sectional metal bands (3M, USA) and wedge (Mina, Iran) were used for restoration. The cavity was etched using 37% phosphoric acid gel (Kimia, Iran) for 20 seconds. The etchant was washed out with water. The enamel part of the cavity was dried with air, and the dentin was dried with a cotton pellet. The bonding (3M, USA) was applied to the cavity using an applicator (Andent, China) according to the manufacturer’s instructions and light-cured for 40 seconds. A flowable composite resin (VOCO-X-tra base, Germany) with a maximum thickness of 4 mm was placed at the bottom of the cavity and light-cured for at least 40 seconds. Then a packable composite resin layer (VOCO-X-TRF FILL, Germany) with a maximum thickness of 4mm was placed on the occlusal surface and light-cured for at least 40 seconds. The occlusion was checked using an articulation paper (Hanel, Germany) and if necessary, adjusted with a polishing bur (Tizkavan, Tehran, Iran).

### Data analysis

After collecting the completed questionnaires and checklists, the data were coded and entered into SPSS 20. The means, standard deviations, frequencies, and percentages were used to analyze descriptive data. The comparison of the frequencies of demographic information in the two groups was carried out using the chi-squared test and Fisher’s exact test. Mann-Whitney test was used to compare the satisfaction of parents and children between the two groups. The frequencies and percentages of answers to the questions were used to show the opinions of parents and children in the treatment session and follow-up sessions. Fisher’s exact test was used to compare the radiographic success of the treatment between composite resin restoration and SSC restoration in follow-up visits. The significance level was set at p< 0.05.

## Results

This experimental study was performed on 70 teeth in 44 children with 4-7 years of age with a mean age of 5.29± 1.2 years in both SSC and composite resin groups over one year. According to Table 1, the demographic characteristics of the children were equally distributed in the two groups. The tooth type, jaw type, and the level of cooperation of children in two groups were homogeneous.

**Table 1:** Demographic data and type of teeth, the jaw, and the level of cooperation of children participating in the two groups

Variable		Study groups				*p* Value ^1^
		Composite		SSC*		
		No.	Percentage	No.	Percentage	
Gender	Female	11	36.7%	14	35.0%	0.964
	Male	19	63.3%	26	65.0%	
	Total	30	100%	40	100%	
Father’s educational level	Some high school education	13	32.5%	10	33.3%	0.553
	High school graduate	6	15.0%	5	16.7%	
	BA	16	40.0%	8	26.7%	
	>MA	5	12.5%	7	23.3%	
	Total	30	100%	40	100%	
Father’s occupation	Unemployed	2	5.0%	0	0.0%	0.173
	Housewife	4	10.0%	0	0.0%	
	Government employee	10	25.0%	8	26.7%	
	Self-employed	24	60.0%	22	73.3%	
	Total	30	100%	40	100%	
Mother’s educational level	Some high school education	4	10.0%	3	10.0%	0.657
	High school graduate	20	50.0%	14	46.7%	
	BA	11	27.5%	6	20.0%	
	>MA	5	12.5%	7	23.3%	
	Total	30	100%	40	100%	
Mother’s occupation	Unemployed	0	0.0%	1	3.3%	0.535
	Housewife	26	65.0%	21	70.0%	
	Government employee	5	12.5%	4	13.3%	
	Self-employed	9	22.5%	4	13.3%	
	Total	30	100%	40	100%	
Tooth type	D	15	50.0%	22	55.0%	0.682
	E	15	50.0%	18	45.0%	
	Total	30	100%	40	100%	
Jaw type	Maxilla	17	56.7%	29	72.5%	0.167
	Mandible	13	43.3%	11	27.5%	
	Total	30	100%	40	100%	
Cooperation level according to Frankl’s scale	3	18	60.0%	20	50.0%	0.278
	4	12	40.0%	20	50.00%	
	Total	30	100%	40	100%	

* SCC: Stainless steel crown

Table 2 shows the frequency distribution and comparison of children and their parents’ responses to the questionnaire concerning the satisfaction with restoration’s color in the treatment session in the two groups. The parents were not satisfied with the color of SSC restoration compared to the parents in the composite resin group (p< 0.001), while this difference was not observed in children (p= 0.246).

**Table 2:** Frequency distribution and comparison of parents and children’s answers to the questions on the questionnaire concerning satisfaction with tooth restoration color in the treatment session in the two groups

Questions on the questionnaire		Study groups (number and percentage)						*p* Value
		SSC*			Composite resin			
		-1	0	1	-1	0	1	*p* < 0.001
Parents	Are you satisfied with the appearance of your child’s restoration?	7	3	30	0	0	30	
		(17.5)	(7.5)	(75.0)	(0.0)	(0.0)	(100.0)	
	Do you believe your child likes his/her restoration’s color?	4	12	24	0	0	30	
		(10.0)	(30.0)	(60.0)	(0.0)	(0.0)	(100.0)	
	Do you believe the restorative procedure was easy for your child?	10	7	23	10	1	19	
		(25.0)	(17.5)	(57.5)	(33.3)	(3.3)	(63.3)	
	Do you believe that the restoration’s color has positively affected your child’s self-confidence?	14	7	19	3	1	26	
		(35.0)	(17.5)	(47.5)	(10.0)	(3.3)	(86.7)	
	Do you believe that the restoration’s color has positively affected your child’s toothbrushing habit?	14	7	19	4	2	24	
		(35.0)	(17.5)	(47.5)	(13.3)	(6.7)	(80.0)	
	Was your child’s restoration cost-effective?	2	7	31	0	2	28	
		(5.0)	(17.5)	(77.5)	(0.0)	(6.7)	(93.3)	
	Do you believe your child’s restoration is durable?	2	13	25	5	11	14	
		(5.0)	(32.5)	(62.5)	(16.7)	(36.7)	(46.7)	
Parents’ satisfaction with the restoration’s color in the treatment session		53	56	171	22	17	171	
		(19.0)	(20.0)	(61.0)	(10.5)	(8.5)	(81.0)	
Children	Do you like the color of your new tooth?	4	3	33	0	2	28	*p* = 0.246
		(10.0)	(7.5)	(82.5)	(0.0)	(6.7)	(93.3)	
	Are you happy with your new tooth?	2	6	32	0	2	28	
		(5.0)	(15.0)	(80.0)	(0.0)	(6.7)	(93.3)	
	Were you comfortable when your tooth was being restored?	8	7	25	5	4	21	
		(20.0)	(17.5)	(62.5)	(16.7)	(13.3)	(70.0)	
Children’s satisfaction with the restoration’s color in the treatment session		14	16	90	5	8	77	
		(12.0)	(13.0)	(75.0)	(6.0)	(9.0)	(85.0)	

(Scoring the questionnaire’s answers: positive effect: 1, negative effect: -1, ineffective: 0

* SCC: Stainless steel crown

Table 3 shows the frequency distribution and comparison of answers of children and their parents to the questions related to the satisfaction with the restoration color in follow-up visits in the two groups. The children were not satisfied with the color of SSC restorations compared to the composite resin group (p< 0.001). However, this difference was not seen in parents (p= 0.246).

**Table 3:** Frequency distribution and comparison of parents and children’s answers to the questions of two questionnaires: satisfaction with the color of restoration and the effect of tooth color on the daily living conditions of the child in the follow-up visits in the two groups

Questions on the questionnaire		Follow-up (month)	Study groups (number and percentage)						*p* value
			SSC*			Composite resin			
			-1	0	1	-1	0	1	*p* > 0.05
Parents	Are you satisfied with the appearance of your child’s restoration?	12	9	2	29	0	1	29	
			22.5%	5.0%	72.5%	(0.0)%	(3.3)%	(96.7)%	
	Does your child like his/her restoration’s color?	12	3	1	36	0	1	29	
			7.5%	2.5%	90.0%	(0.0)%	(3.3)%	(96.7)%	
	Has the color of the tooth positively affected your child’s toothbrushing habit?	12	7	5	28	8	2	20	
			17.5%	12.5%	70.0%	26.7%	6.7%	66.7%	
	Has your child ever had a problem due to his/her tooth color?	12	0	5	35	0	2	28	
			(0.0)%	(12.5)%	(87.5)%	(0.0)%	(6.7)%	(93.3)%	
	Has the tooth color positively affected your child’s attitude toward dentistry?	12	0	10	30	17	2	11	
			(0.0)%	(25.0)%	(75.0)%	(56.7)%	(6.7)%	(36.7)%	
	Has the tooth color positively affected your child’s relationships with his/her peers?	12	1	9	30	5	6	19	
			2.5%	22.5%	75.0%	16.7	20.0%	63.3%	
Parents’ satisfaction with the restoration’s color in the treatment session		12	20	32	188	30	14	136	
			8%	13%	79%	16.7%	7.8%	75.5%	
Children	Do you like the color of your restored tooth?	3	3	0	37	0	0	30	*p*<0.001
			7.5%	0.0%	92.5%	0.0%	0.0%	100.0%	
		6	3	0	37	0	0	30	
			7.5%	0.0%	92.5%	0.0%	0.0%	100.0%	
		12	4	0	36	0	0	30	
			10.0%	0.0%	90.0%	0.0%	0.0%	100.0%	
	Has anybody ever asked you about your tooth color?	3	23	0	17	3	0	27	
			57.5%	0.0%	42.5%	10.0%	0.0%	90.0%	
		6	22	0	18	3	0	27	
			55.0%	0.0%	45.0%	10.0%	0.0%	90.0%	
		12	19	0	21	0	0	30	
			47.5%	0.0%	52.5%	0.0%	0.0%	100.0%	
	Have you ever been laughed at because of your tooth color?	3	5	0	35	0	0	30	
			12.5%	0.0%	87.5%	0.0%	0.0%	100.0%	
		6	3	0	37	0	0	30	
			7.5%	0.0%	92.5%	0.0%	0.0%	100.0%	
		12	3	2	35	0	0	30	
			7.5%	5.0%	87.5%	0.0%	0.0%	100.0%	
	Has your tooth color ever prevented you from smiling and laughing?	3	0	0	40	0	0	30	
			0.0%	0.0%	100.0%	0.0%	0.0%	100.0%	
		6	0	0	40	0	0	30	
			0.0%	0.0%	100.0%	0.0%	0.0%	100.0%	
		12	0	2	37	0	0	30	
			0.0%	7.5%	92.5%	0.0%	0.0%	100.0%	
	Is the color of your tooth important for you after restoration?	3	28	2	10	8	6	16	
			70.0%	5.0%	25.0%	26.7%	20.0%	53.3%	
		6	29	2	9	8	6	16	
			72.5%	5.0%	22.5%	26.7%	20.0%	53.3%	
		12	27	4	9	8	6	16	
			67.5%	10.0%	22.5%	26.7%	20.0%	53.3%	
Children’s satisfaction with the restoration’s color in the treatment session		3	59	2	139	11	6	133	
			29.5%	1%	69.5%	7.4%	4%	88.6%	
		6	57	2	141	11	6	133	
			28.5%	1%	70.5%	7.4%	4%	88.6%	
		12	53	9	138	8	6	136	
			26.5%	4.5%	69%	5.4%	4%	90.6%	

* SCC: Stainless steel crown

(Scoring the questionnaire answers: positive effect: 1, negative effect: 1-, ineffective: 0)

Table 4 presents the evaluation and comparison of variables related to clinical and radiographic criteria of treated teeth in the two groups after 12 months. The frequency of clinical success was 95% in the SSC group and 96.7% in the composite resin group. The frequency of radiographic success was 87.5% in the SSC group and 100% in the composite resin group.

**Table 4:** Comparison of the success rates of clinical and radiographic criteria in the two study groups in the 12-month follow-ups

	Variable		Study groups				*p* Value ^1^
			SSC*		Composite resin		
			No.	Percentage	No.	Percentage	
Clinical criteria	Loss of the restoration	No	40	100.00%	30	100.00%	-
	Recurrent caries	No	40	100.00%	30	100.00%	-
	Marginal gap	No	39	97.50%	29	96.70%	0.57
		Yes	1	2.50%	1	3.30%	
	Occlusal contact	Yes	40	100.00%	30	100.00%	-
	Proximal contact	No	1	2.50%	0	0.00%	0.654
		Yes	39	97.50%	30	100.00%	
	Total clinical failure		2	5.0%	1	3.30%	0.749
Radiographic	criteria	Furcal radiolucency	No	40	100.0%	30	100.0%
	Apical radiolucency	No	38	95.0%	30	100.0%	0.503
		yes	2	5.0%	0	0.0%	
	PDL widening	No	37	92.5%	30	100.0%	0.225
		yes	3	7.5%	0	0.0%	
	Follicle involvement	No	40	100.0%	30	100.0%	-
	Internal root resorption	No	40	100.0%	30	100.0%	-
	Pathological external root resorption	No	40	100.0%	30	100.0%	-
	Coronal recurrent caries	No	40	100.0%	30	100.0%	-
	Total radiographic failure		5	12.5%	0	0.0%	0.061

* SCC: Stainless steel crown

Significance level calculated based on Fisher’s exact test

## Discussion

This study showed that the color of the restoration in the treatment session is important for parents, and after a year, it was not very important for them. However, in children, it was the opposite. The children’s friends see the different colors of the tooth in their mouth, and their reaction might affect and change children’s idea over time. Nevertheless, perceptions of tooth esthetics would affect individuals’ social and psychological well-being and would reflect in their behavior and self-confidence [ 15 ]. Currently, parents’ demands, considering esthetic restorations for their children, are increasing and public awareness about esthetics is growing [ 16 ]. Mathew *et al.* [ 16 ] examined parental satisfaction with SSC restoration at 6, 12, 18, 24 and 36 months, and reported that only 40% of parents and 53.3% of children were satisfied with SSC restoration, and their satisfaction did not change over time. Nonetheless, 100% of parents and children were satisfied with zirconia restorations [ 16 ]. Utami *et al.* [ 17 ] reported that 90% of children had positive attitudes toward SSC restoration, and they accepted SSC. However, only 53.5% of parents accepted SSC restorations [ 17 ]. Akhlaghi *et al.* [ 14 ] evaluated the attitudes of parents and children towards SSC and found that 81.3% of children were satisfied with the appearance of the SSCs, and 77.6% were happy with the metal teeth. However, only 30% of parents were satisfied with the appearance of the crown [ 14 ]. According to a study by Zimmerman *et al.* [ 18 ], the main concerns of parents about metal crowns were related to esthetics, cost, toxicity, and durability of these restorations, respectively. The reason for the different reactions of children and parents to the color of the restored tooth might be that improving the function and eliminating children’s pain leads to a sense of satisfaction in the parents, which later decreases their sensitivity and attention to the tooth color. However, the child suffers from possible psychological effects due to the dark color of SSC, and the longer the SSC is in the mouth, the more unpleasantly the child experiences the daily events due to the metallic color of the crown.

In the current study, the clinical and radiographic success rate of composite resin and SSC restoration in the one-year follow-up was similar, which could be due to employment of bulk-fill composite resin, combined use of flow and packable composite resin, and the use of orthodontic bands instead of a matrix tape. Banding was used to save time and eliminate the time-consuming steps to place a matrix tape to prepare the tooth for the composite resin restoration. The band had a proper gingival adaptation and an interproximal contour. Orthodontic braces have formerly been used to restore class II cavities in children [ 19 ]. Alyahya *et al.* [ 20 ] reported that the durability of composite resin restorations in class II cavities did not differ between 41.3 months and 45.6 months [ 20 ]. However, Zahdan *et al.* [ 21 ] retrospectively evaluated the durability of SCCs and multi-surface composite resin restorations, and reported that SCCs had higher durability than multi-surface composite resin restorations, and the clinical success in SSC was 98.5%, with 79% in flowable composite resin. Chen *et al.* [ 22 ] evaluated the satisfaction of children treated with SSC and composite resin under general anesthesia. The results showed that the composite resin was not different from SSC in terms of durability, margin problems, and recurrent caries in 6- and 18-month follow-ups. However, in a 24-month follow-up, SSC was superior to composite resin restoration [ 22 ]. In this study, the bulk-fill composite resin was used, which facilitates and increases the speed of tooth restoration in children. These composite resins can be cured at depths &gt;4 mm while exerting slight shrinkage stress to the cavity walls [ 23 ]. High curing depths of these composite resins reduces the number of composite resin layers, decreases the curing time, which is especially important in pediatric dentistry and subsequently reduces the risk of contamination, and improves the cooperation between child and dental practitioner [ 24 ]. The use of flowable composite resin as a liner and then the use of packable composite resins effectively reduce microleakage in the gingival margins [ 25 ]. Flowable composite resins have been proposed as a cavity liner with well adaptation to the cavity microstructure irregularities, improved marginal adaptation, reduced microleakage; all these advantages leads to lower rates of recurrent caries and restoration failure [ 26 ].

Since the age of esthetic appraisal in children has decreased and children in the modern world pay attention to their appearance from 3 to 5 years of age, dentists should also pay attention to their opinion in this task about esthetic and mind their opinion in choosing the restoration color. One of the limitations of this study was finding children who were eligible for the study, who had eight vital primary molars with extensive multi-surface caries, and the restoration of all eight teeth with composite or SSC could have the maximum impact on the child’s living conditions. However, in this study, children with at least one primary molar up to a maximum of eight primary molars with extensive and multi-surface caries were included.

Further studies are suggested to evaluate more realistic and broader dimensions of the effects of SSC metallic color on daily living of children by consulting children’s psychologists. Moreover, since a one-year period is short time for evaluating a restoration in the patient’s oral cavity, subsequent studies can be designed with longer follow-up periods.

## Conclusion

Although the color of restoration is not important for children in their first encounter, they were significantly more satisfied with the white color of composite resin compared to the metallic color of SSC over time. Therefore, it seems that the color of tooth restoration, as one of the esthetic criteria, is considered even at young ages. On the other hand, parents who reacted to the color of their child’s restoration in the same session and were more satisfied with the white color of the composite restoration right after seeing the tooth than the SSC metallic color did not show any sensitivity to the color of their children’s teeth after a year. The application of bulk-fill flowable and bulk-fill packable composite resin resulted in clinical and radiographic success similar to SSC for restoring primary molar teeth.

## Acknowledgement

We thank Mr. A. Nekoui for his statistical comments. This paper is based on the corresponding author’s thesis (specialty thesis #199) carried out at the Dental School of Kerman University of Medical Sciences and supervised by Dr. R. Shojaeipour.

## Conflicts of Interest

The authors declare that they have no conflict of interest. 
